# Depleting PTOV1 sensitizes non-small cell lung cancer cells to chemotherapy through attenuating cancer stem cell traits

**DOI:** 10.1186/s13046-019-1349-y

**Published:** 2019-08-06

**Authors:** Zhiqiang Wu, Zhuang Liu, Xiangli Jiang, Zeyun Mi, Maobin Meng, Hui Wang, Jinlin Zhao, Boyu Zheng, Zhiyong Yuan

**Affiliations:** 1 Department of Radiation Oncology, Tianjin Medical University Cancer Institute & Hospital, Key Laboratory of Cancer Prevention and Therapy, National Clinical Research Center for Cancer, Tianjin’s Clinical Research Center for Cancer, Tianjin, 300060 China; 2 Department of Thoracic Medical Oncology, Tianjin Medical University Cancer Institute & Hospital, Tianjin, 300060 China; 3 Department of Biochemistry and Molecular Biology, College of Basic Medical Science, Tianjin Medical University, Tianjin, 300070 China

**Keywords:** Non-small cell lung cancer, PTOV1, Chemotherapy, Cancer stem cell, β-Catenin

## Abstract

**Background:**

Prostate tumor over expressed gene 1 (PTOV1) has been reported as an oncogene in several human cancers. However, the clinical significance and biological role of PTOV1 remain elusive in non-small cell lung cancer (NSCLC).

**Methods:**

The Cancer Genome Atlas (TCGA) data and NCBI/GEO data mining, western blotting analysis and immunohistochemistry were employed to characterize the expression of PTOV1 in NSCLC cell lines and tissues. The clinical significance of PTOV1 in NSCLC was studied by immunohistochemistry statistical analysis and Kaplan–Meier Plotter database mining. A series of in-vivo and in-vitro assays, including colony formation, CCK-8 assays, flow cytometry, wound healing, trans-well assay, tumor sphere formation, quantitative PCR, gene set enrichment analysis (GSEA), immunostaining and xenografts tumor model, were performed to demonstrate the effects of PTOV1 on chemosensitivity of NSCLC cells and the underlying mechanisms.

**Results:**

PTOV1 is overexpressed in NSCLC cell lines and tissues. High PTOV1 level indicates a short survival time and poor response to chemotherapy of NSCLC patients. Depleting PTOV1 increased sensitivity to chemotherapy drugs cisplatin and docetaxel by increasing cell apoptosis, inhibiting cell migration and invasion. Our study verified that depleting PTOV1 attenuated cancer stem cell traits through impairing DKK1/β-catenin signaling to enhance chemosensitivity of NSCLC cells.

**Conclusion:**

These results suggest that PTOV1 plays an important role in the development and progression of human NSCLC and PTOV1 may serve as a therapeutic target for NSCLC patients.

**Electronic supplementary material:**

The online version of this article (10.1186/s13046-019-1349-y) contains supplementary material, which is available to authorized users.

## Background

Lung cancer is the most common diagnosed cancer worldwide [1], and non-small cell lung cancer (NSCLC) accounts for approximately 85% of the disease [2]. Chemotherapy is an important component of treatment for all stages of NSCLC. Patients with early-stage NSCLC received curative surgery benefit with improved survival rates when adjuvant platinum-based chemotherapy is given [3, 4]. However, most NSCLC patients (~ 77%) are diagnosed at late stage, with approximately 55% of patients having metastatic disease at diagnosis. For these patients, chemotherapy is the fundamental treatment and is critical in determining their survival and quality of life. Platinum-based therapy is the mainstay of chemotherapy for NSCLC and is usually given in combination with other agent, such as docetaxel [5]. Although chemotherapy improves outcomes of NSCLC patients, chemoresistance continues to pose a significant challenge in the management of the disease.

Cancer stem cells (CSCs) are tumor cells that possess the principal properties of self-renewal, clonal tumor initiation and long-term repopulation capacity [6, 7]. CSCs have been reported in most types of human cancers, including NSCLC. It was reported that CD133^+^ lung cancer cells represented the cancer stem cell population, which were able to grow indefinitely as tumor spheres in serum-free medium containing EGF and bFGF and tumorigenic [8]. Moreover, expression of pluripotency factors, such as Oct4 and Nanog, also proved to enhance lung cancer stem-like properties [9, 10]. CSCs are a rare population but contribute significantly to tumor initiating, recurrence and resistance to radio- and chemo-therapy [11, 12]. Giulia Bertolini et al. reported that lung cancer CD133^+^ cells display stem-like features and resistant to cisplatin [13]. Hence, targeting CSCs could increase chemsensitivities in NSCLC.

Prostate Tumor Overexpressed 1 (PTOV1) was initially identified during screening for genes overexpressed in prostate cancer [14]. PTOV1, a 46 KDa protein with two repeated PTOV homology blocks of 151 and 147 amino acids, is encoded by a 12-exon gene located on a region of chromosome 19 (19q13) [14]. Overexpression of PTOV1 has been found in multiple cancers such as prostate cancer, breast cancer, and liver cancer [15]. And upregulation of PTOV1 associated with increased proliferation, retinoic acid resistance, aggressiveness, migration and poor prognosis [16–20]. Strikingly, Yanmei et al. recently found that PTOV1 contributes to maintenance of the CSCs by activation of the Wnt/β-catenin pathway in breast cancer [21]. These findings prompt us to ask whether PTOV1 could be a prognosis factor and target for increasing chemosensitivity in NSCLC.

In this study, we analysed the PTOV1 expression in public available data and clinical samples of NSCLC patients. The data uncovered that PTOV1 is upregulated and as an independent and poor prognosis factor in NSCLC. Additionally, our results showed that depleting PTOV1 sensitizes lung cancer cell lines to chemotherapeutic drugs, cisplatin and docetaxel in vitro and in vivo, through attenuating cancer stem cell traits. Our results may provide a novel potential target to overcome chemo-resistance in patients with NSCLC.

## Methods

### Patient information and tissue specimens

Patient consent was informed and written. Patient consent and approval from the Institutional Research Ethics Committee of Tianjin Cancer Institute and Hospital were obtained for the use of the clinical materials for research purposes. A total of 150 paraffin-embedded, archived NSCLC adenocarcinoma samples, which were histopathologically and clinically diagnosed at the Tianjin Medical University Cancer Institute and Hospital between December 2012 and January 2014 were used. Patients were followed up until April 1, 2018 in our study. Clinical information of the samples is summarized in Additional file 1: Table S1.

### Bioinformatics analysis

RNA array datasets, including GSE10072 [22], GSE19804 [23], GSE32863 [24], GSE19188 [25], from the NCBI/GEO database (https://www.ncbi.nlm.nih.gov/gds/)and RNA seq data from the lung Pan-Cancer study of The Cancer Genome Atlas (TCGA) database (https://cancergenome.nih.gov/) were used to analyze the expression of PTOV1 in normal and tumor lung tissues. GEO/ GSE54712 [26] and GEO/GSE64999 [27] datasets were used to investigate the expression of PTOV1 in undifferentiated and serum induced-differentiated spheroids cells. The online Kaplan–Meier analysis of the survival of all lung cancer patients or the subgroup patients accepted chemotherapy with different PTOV1 expression levels was used the Kaplan–Meier Plotter (http://kmplot.com/analysis/) [28]. Gene Set Enrichment Analysis (GSEA) was performed by gsea-3.0.jar (http://software.broadinstitute.org/gsea/index.jsp) using the NCBI/GEO/GSE3141 [29] dataset.

### Cell culture, siRNA, plasmids and stable cell lines construction

NSCLC cell lines, including A549, H460, H1299, H292, Lung bronchus epithelial cell line BEAS-2B and 293FT were obtained from cell banks of Shanghai Institutes of Biological Sciences (Shanghai, China). Calu-3, H1975 and H520 were obtained from ATCC (Manassas, VA, USA). PC-9 was obtained from European Collection of Authenticated Cell Cultures. All cell lines used were authenticated and confirmed to be mycoplasma negative by MycoAlertTM Mycoplasma Detection Kit (Lonza, Basel, Switzerland). NSCLC cell lines were cultured in RPMI 1640 medium (Gibco, Grand Island, NY, USA) and 293FT cells were cultured in DMEM medium (Gibco), supplemented with 10% FBS (BI), 1% Non-essential amino acids, 2 mM L-glutamine, 100 U/ml penicillin, and 100 μg/ml streptomycin (Gibco). BEAS-2B was cultured in BEGM medium (Lonza). Cells were maintained in a humidified atmosphere of 5% CO_2_ at 37 °C.

Negative control (NC) and DKK1 siRNAs were purchased from RiboBio (RiboBio Co., Ltd). The target sequence of siDKK1 was 5′-AAUGGUCUGGUACUUAUUCC-3′.

Two Single-guide RNAs (SgRNAs) targeting PTOV1 were synthesized by AuGCT Biotechnology Co., Ltd. (Beijing, China) and further annealed and ligated into the lentiCRISPR V2 vector [30]. The target sequences were: Sg1, 5′-CGGACGGCGCGC ACCACGAG-3′ and Sg2, 5′-CCCGGCGCCGGAGCGGTACG-3′.

Plasmids, lentiCRISPR V2-PTOV1-Sg1/2, pSPAX2 and pMD.2G, were co-transfected into 293FT cells to pack lentivirus. The virus containing medium were harvested and infected H460 and Calu-3 cells for 48 h. Then the infected cells were selected with puromycin (1 μg/ml, Sigma-Aldrich, St. Louis, MO, USA) to construct stable cell lines with PTOV1 depleted. pLVX-neo-eGFP was packed into virus to infect the cells for constructing eGFP tagged cells.

### Western blotting

Western blotting was performed as previously reported [31]. The primary antibodies against PTOV1 (SAB1301047, Sigma), GAPDH (sc-365,062, Santa Cruz), β-catenin (610,153, BD Biosciences), Caspase-3 (#9662), Cleaved-caspase-3 (#9661), Bcl-2 (#2876, Cell Signaling Technology), LaminB1 (YM3036, Immunoway) and DKK1 (21112–1-AP, Proteintech) were used.

### Quantitative real-time PCR

Total RNAs were extracted by using the Trizol Reagent (Invitrogen, Carlsbad, CA, USA) according to the manufacturer’s instruction. Two microgram of total RNA from each sample were reverse transcribed into cDNA. Quantitative Real-Time PCR was carried out using Trans Start Top Green qPCR Super Mix (Transgene, Beijing, China) with a CFX96™ Real-Time System (BIO-RAD, Hercules, CA, USA). The relative mRNA levels were analyzed by the 2^(−ΔΔCt)^ method with GAPDH as a control. The primers sequences were listed in Additional file 1: Table S2.

### CCK-8 assay

Cells were seeded in 96-well plates (5 × 10^3^ cells/well). After drug treatment at the indicated doses for 48 h, cell viability were measured using the Cell Counting Kit-8 (CCK8, Biyuntian, Beijing, China) assay. Briefly, discard the medium, add diluted CCK-8 solution (10% in medium) to each well and incubated further for 1.5 h. The optical density value was measured at a wavelength of 450 nm. The following formula was used to calculate the cell survival rate: Cell survival rate (%) = [(As-Ab) / (Ac-Ab)] × 100%.

### Colony formation assay

Cells were seeded into a 24-well plate (5 × 10^4^ cells/well) and cultured in RPMI 1640 medium, which contained drug at the indicated doses, in a humidified atmosphere of 5% CO_2_ at 37 °C for 48 h. Then colonies were fixed and stained with crystal violet.

### Flow cytometry analyses of cell apoptosis, cleaved caspase-3 and CD133^+^ cells

Cell apoptosis was analyzed using an Annexin V-FITC Apoptosis Detection kit (BD, bioscience) according to the manufacturer’s instruction. Briefly, cells were trypsinized and seeded in 60-mm culture dish. After treatment with cisplatin (10 μg/ml) for 12 h or docetaxel (0.01 μg/ml) for 24 h, cells were harvested, washed twice with PBS and stained with Annexin V-FITC and propidium iodide (PI) for 15 min.

Cleaved caspase-3 were analyzed using Alexa Fluor® 488-conjugated cleaved caspase-3 (Asp175) (D3E9) antibody (1:500, #9603, Cell Signaling Technology) with Alexa Fluor® 488-conjugated rabbit (DA1E) mAb IgG XP® Isotype antibody (#2975, Cell Signaling Technology) as a reference control. CD133 was analyzed by PE-conjugated anti-human CD133 antibody (1:50, 372,804, BioLegend) with PE-conjugated Mouse IgG1, κ isotype ctrl (FC) antibody (400,114, BioLegend) as a reference control.

Each assay had been performed for three times.

### Immunofluorescence staining

Cells were trypsinized, seeded on coverslips (Thermo Fisher Scientific) incubated overnight. The cells were then washed with PBS, fixed with 4% paraformaldehyde for 1 min and then with prechilled methanol for another 15 min at room temperature, permeabilized with 0.2% Triton X-100 in PBS (PBS-T) for 15 min, blocked with 10% BSA in PBS-T for 30 min, and incubated anti-β-catenin (610,153, BD bioscience) over night at 4 °C. Cells were then incubated with TRITC-conjugated secondary antibody (Jackson Immuno-Research Laboratories) for 1 h at room temperature. Coverslips were mounted with ProLong Diamond Anti-fade reagent with DAPI (Invitrogen). Gray level images were acquired under a laser scanning microscope (Axio Imager.Z2, Carl Zeiss Co. Ltd.).

### Tumor sphere formation assay

Cells were trypsinized and seeded at a density of 1 × 10^3^ cells/well for H460 or 3 × 10^3^ cells/well for Calu-3 in six-well ultra-low attachment culture plates (Corning Life Sciences) and cultured in DMEM/F12 serum-free medium with 20 ng/ml epidermal growth factor (EGF), 10 ng/ml basal fibroblast growth factor (bFGF), 5 μg/ml insulin, 0.4% bovine serum albumin (BSA), 2% B-27 Supplement (Research and Development). The spheres were then photographed and counted.

### Immunohistochemistry

Immunohistochemistry was performed as described previously [32]. Anti-PTOV1 (SAB1301047, Sigma), anti-Ki67 (273019-I-AP, Proteintech), anti-cleaved-caspase-3(#9661, Cell Signaling Technology), anti-β-catenin (610,153, BD) antibodies were used. The sections were reviewed independently by two blinded observers and scored based on both the proportion of positively stained tumor cells and the intensity of the staining. The proportion of tumor cells was graded as follows: 0 (no positive tumor cells), 1 (< 10% positive tumor cells), 2 (10–50% positive tumor cells), 3 (50–75% positive tumor cells), and 4 (> 75% positive tumor cells). The intensity of staining was recorded as follows: 1 (no staining), 2 (weak staining, light yellow), and 3 (strong staining, brown). The staining index was calculated as the proportion of positive cells × staining intensity score.

### Tumor xenografts

The animal experimental protocol was approved by the Institutional Animal Care and Use Committee of Tianjin Medical University. Female BALB/c nude mice (5-6 weeks old) were purchased from Nanjing Biomedical Research Institute of Nanjing University (NBRI) and housed in specific pathogen-free facilities on a 12 h light/dark cycle and separated randomly into four groups (5 for each group). Then, H460-Vector and H460-Sg2 cells (3 × 10^6^) in 100μl of basic RPMI 1640 medium subcutaneously injected into nude mice. Tumor volume was monitored every 3 days with a caliper and calculated by the formula: 0.5 × length × width^2. When tumor volume was approximately 200 mm^3^ (~ 1 weeks after cells inoculation), the mice were injected intraperitoneally with saline (control groups) or docetaxel (10 mg/kg, experimental group) every 3 days. After 2–3 weeks, the nude mice were sacrificed and the xenograft tumors were excised, weighed and fixed with 4% formalin for further study.

### Statistical analysis

Statistical analyses were carried out using the SPSS 19.0 statistical software package or GraphPad Prism 6.0 software. The relationship between PTOV1 expression and the clinicopathological characteristics was analyzed by the chi-square test. Survival curves were plotted by the Kaplan-Meier method and compared using the log-rank test. Survival data were evaluated using univariate and multivariate Cox regression analyses. Two-tailed, unpaired Student’s t-test or one-way ANOVA test were used for comparisons between groups for statistical significance. A *p* value < 0.05 was considered statistically significant.

## Results

### PTOV1 is significantly upregulated in NSCLC

**Fig. 1:**
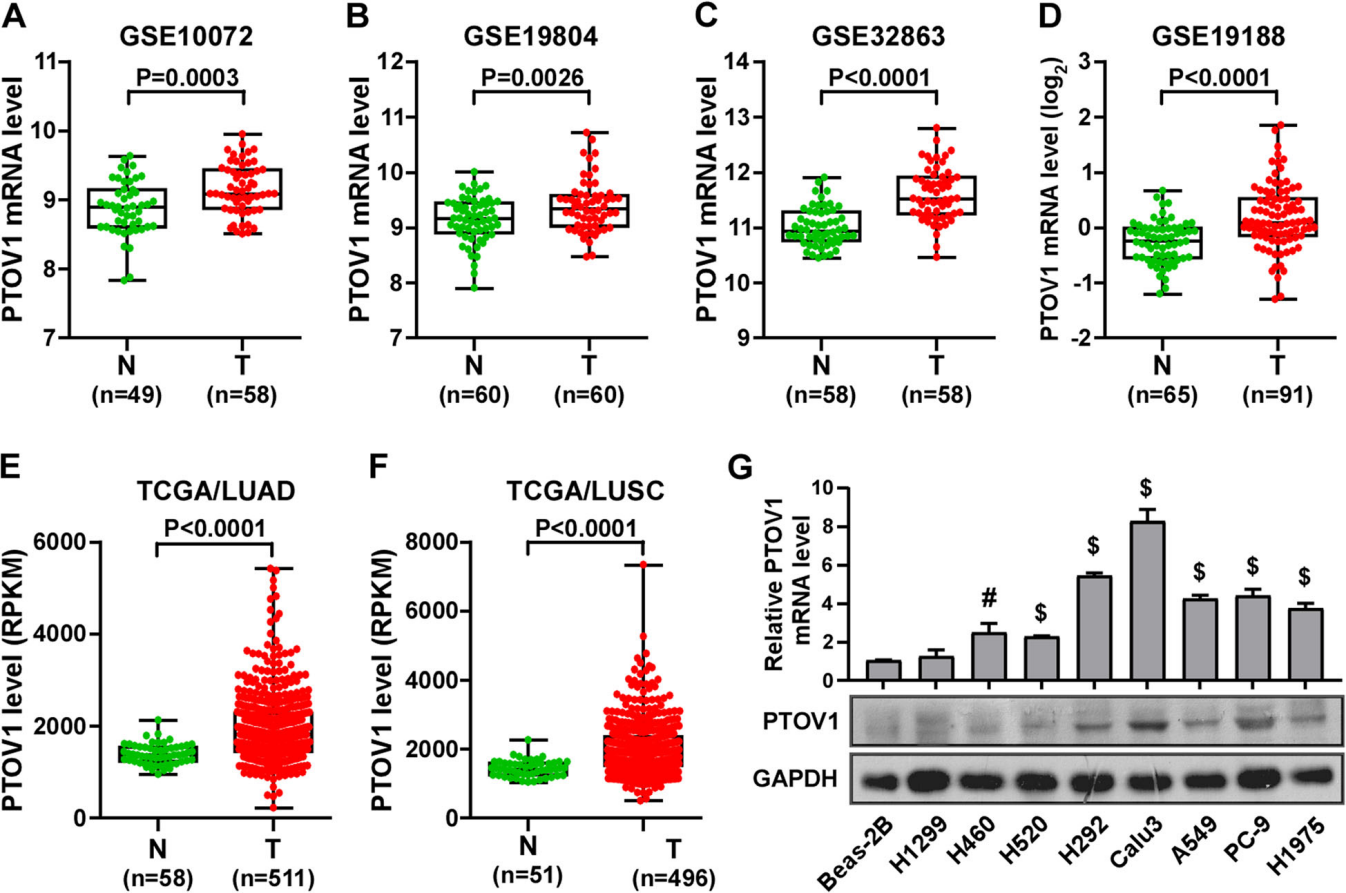
PTOV1 is upregulated in NSCLC**. a-d** Analysis of the expression of PTOV1 mRNA in NSCLC datasets from the GEO database. **e** and **f** Analysis of the expression of PTOV1 mRNA in NSCLC datasets from the TCGA database. **g** Real-time PCR (upper panel) and western blotting (lower panel) analyses of PTOV1 expression in lung epithelial cell line, Beas-2B, and 8 NSCLC cell lines. N, normal tissue. T, tumor tissue. Error bars represent the mean ± SD form 3 independent experiments. *P* values are calculated by two-tailed, unpaired t-test. *, *P* < 0.05; #, *P* < 0.01; $, *P* < 0.001

PTOV1 was reported to be upregulated in kinds of cancer, however there is no systematic analysis of the expression of PTOV1 in NSCLC. First, we explored the NCBI/GEO database (https://www.ncbi.nlm.nih.gov/gds/?term=) to investigate the expression of PTOV1 in NSCLC. Surprisingly and consistently, compared to the normal lung tissues, PTOV1 mRNA was obviously upregulated in NSCLC tumor tissues in all analyzed files (Fig. 1a-d). Data from the TCGA database also showed that PTOV1 levels were dramatically increased in both lung adenocarcinoma (LUAD) and lung squamous cell carcinoma (LUSC) tissues as compared to the normal control (Fig. 1e, f). Moreover, real-time PCR and western blotting analyses showed that PTOV1 mRNA and protein were also heterogeneously upregulated in NSCLC cell lines comparing with the control, Beas-2B cells (Fig. 1g). These all proved upregulation of PTOV1 in NSCLC.

### PTOV1 is an independent and poor prognosis factor of NSCLC patients

**Fig. 2:**
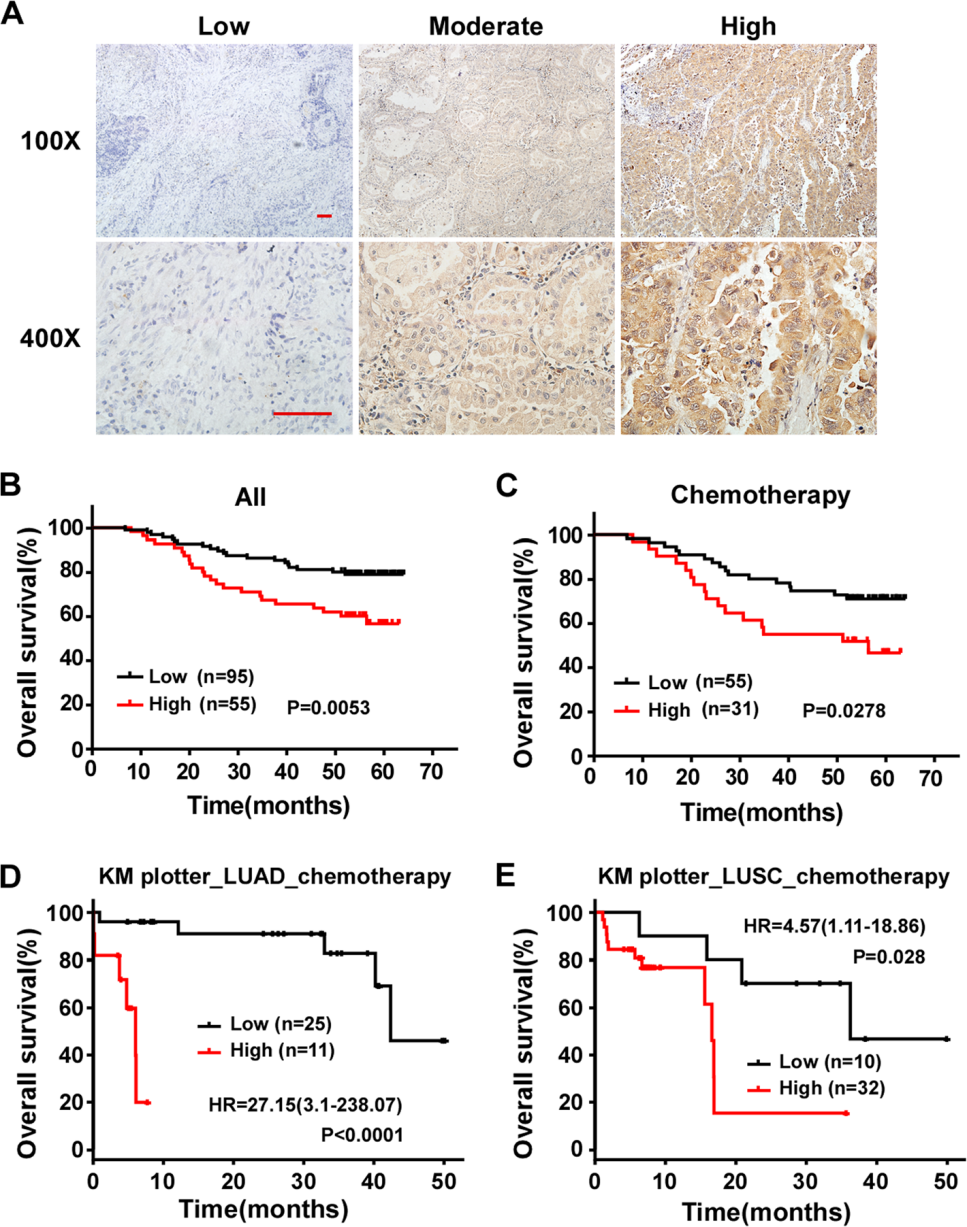
High PTOV1 expression correlates with poor prognosis and chemo resistance in NSCLC patients. **a** Representative pictures of NSCLC tissues with low, modulate and high PTOV1 expression levels analyzed by IHC staining. Scale bars, 50 μm. **b** and **c** Kaplan-Meier analysis of overall survival in all NSCLC patients (**b**) and the subgroup patients received chemotherapy (**c**). **d** and **e** Kaplan-Meier analysis of overall survival in the LUAD (**d**) and LUSC (**e**) subgroup patients received chemotherapy by the Kaplan–Meier Plotter online database. *P* values are calculated by log-rank test

Next, we explored the clinical significance of PTOV1 in NSCLC. IHC staining was performed to exam the expression of PTOV1 in a cohort of 150 paraffin-embedded, archived NSCLC tissues. Most tissues (93.33%) were positively stained with PTOV1. PTOV1 localized at both cytoplasm and nucleus and representative images of different PTOV1 expression level were shown in Fig. 2a. Statistical analysis revealed no correlation between PTOV1 expression and clinicopathological characteristics of patient in this cohort of samples (Additional file 1: Table S1). Univariate Cox regression analysis showed that PTOV1 significantly correlated with patients’ survival status (Regression coefficient = 0.830, *p* = 0.007, Table 1). Multivariate Cox analysis indicated that PTOV1 is an independent prognosis factor of NSCLC with hazard ratio of 2.588 (*p* = 0.002, Table 1). Kaplan–Meier survival analyses showed that NSCLC patients with high PTOV1 had a shorter overall survival time than the ones with low PTOV1 in both all patients and the subgroup received chemotherapy (Fig. 2b, c). Mining data from the Kaplan–Meier Plotter (http://kmplot.com/analysis/index.php?p=service&cancer=lung) and the TCGA database showed that PTOV1 level significantly and negatively associates with overall survival in LUAD, but not LUSC, patients (Additional file 2: Figure S1A and B). However, surprisingly, PTOV1 level significantly correlates with overall survival in subgroup patients who received chemotherapy in both LUAD and LUSC (Fig. 2d and e). Additionally, PTOV1 high expression also associated with poor first progression survival time of LUAD patients from the Kaplan–Meier Plotter database (Additional file 2: Figure S1C). These results suggested that PTOV1 is a poor prognosis factor in NSCLC.

**Table 1:** Prognostic analysis of univariate and multivariate Cox proportional hazards in patients with NSCLC

Characteristics	Univariate analysis		Multivariate analysis		
	p	Regression coefficient (SE)	p	Relative risk	95% confidence interval
PTOV1	0.007	0.830 (0.306)	0.002	2.588	1.398–4.789
Smoke	0.031	0.664 (0.307)	0.477	–	–
Sex	0.006	0.866 (0.312)	0.001	2.896	1.551–5.405
T stage	0.001	0.689 (0.200)	0.050	1.606	1.001–2.578
N stage	< 0.001	0.780 (0.169)	< 0.001	2.161	1.518–3.077
Clinical stage	< 0.001	0.687 (0.153)	0.546	–	–

### Inhibiting PTOV1 increases sensitivity to chemotherapy of NSCLC cells in vitro

**Fig. 3:**
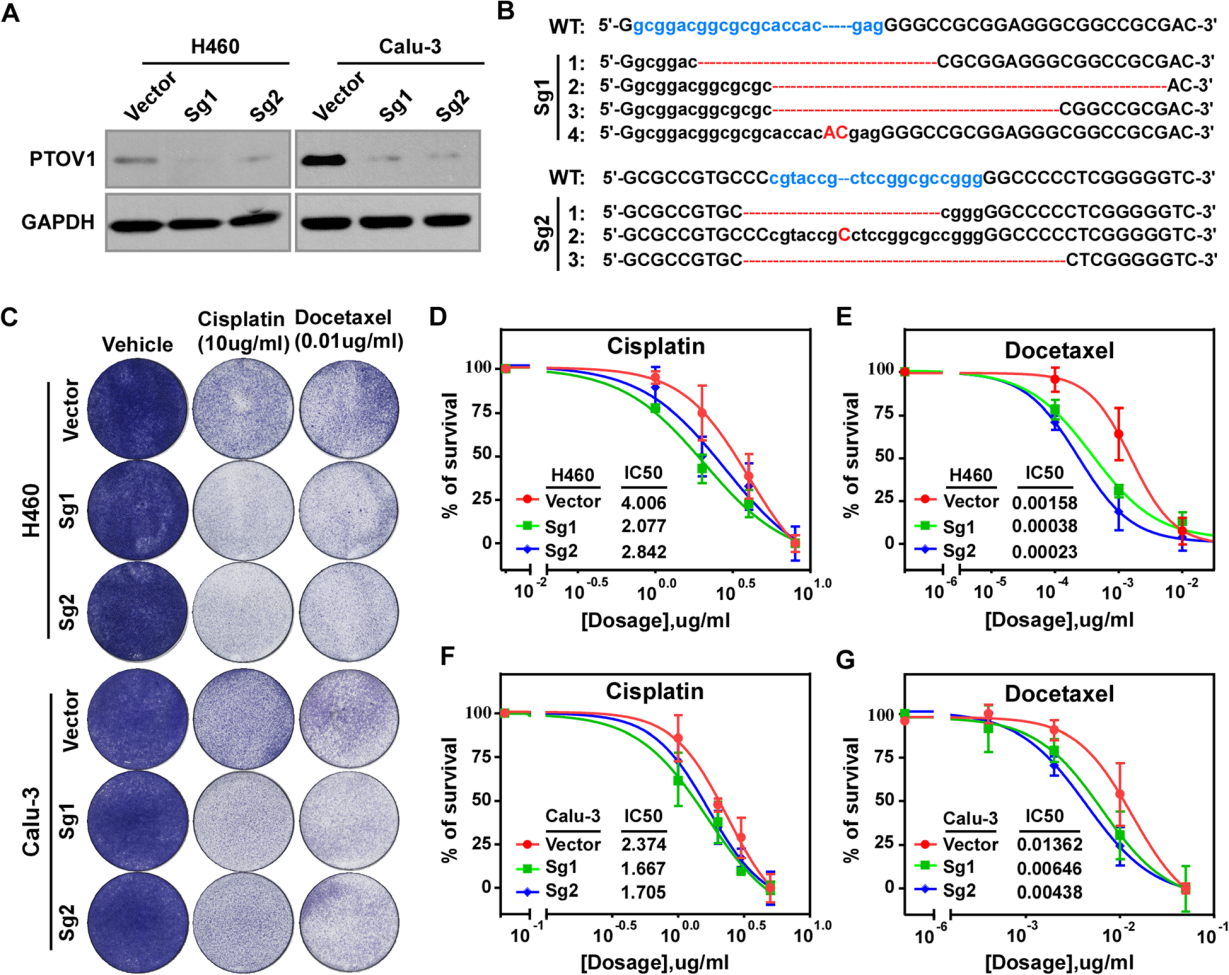
Depleting PTOV1 increases sensitivities to cisplatin or docetaxel of NSCLC cells. **a** Western blotting analyzing the expression of PTOV1 in the indicated cells. **b** Representative sequencing results of different indels introduced by SgRNAs in pooled PTOV1-Sg1 and –Sg2 cells comparing to the wide type (WT) PTOV1 locus. Blue lowercase letters indicate the target sequences of SgRNA 1 and 2. Red dash lines and capital letters are the indels. **c** Colony formation of the indicated cells after treatment with cisplatin or docetaxel. **d-g** IC50s the indicated cells responding to cisplatin or docetaxel determined by CCK-8 assay

As high expression of PTOV1 predicting poor prognosis in NSCLC and the reported oncogenic roles of PTOV1 in types of cancer [17, 21, 33], we presumed that depletion of PTOV1 might benefit NSCLC treatment. To prove that, 2 SgRNAs (named Sg1 and Sg2 respectively) were designed to deplete PTOV1 using CRISPR/Cas9 system in NSCLC cell lines H460 and Calu3. Western blotting analysis proved successful depletion of PTOV1 (Fig. 3a). Amplification of the targeted genomic DNA and sequencing revealed different types of indels that led to silencing of PTOV1 expression (Fig. 3b).

Chemotherapy improves patients’ outcomes and is one main therapeutics for NSCLC. Herein depletion of PTOV1 on the effects of chemotherapy of NSCLC was investigated. Colony formation assay showed that there were less cells survived in PTOV1 Sg1 and Sg2 cells treated with cisplatin and docetaxel, two first-line drugs for NSCLC chemotherapy [34], comparing to the vector cells (Fig. 3c). Cell viability evaluated by CCK-8 assay showed that the 50% inhibitory concentrations (IC50s) of PTOV1 depleted cells responding to cisplatin and docetaxel were much lower than that of the vector control cells (Fig. 3d-g). These data indicated that inhibiting the expression of PTOV1 increases chemosensitivities of NSCLC cells.

### Depleting PTOV1 inhibited migration and invasion and enhanced apoptosis induced by chemotherapy in NSCLC cells

**Fig. 4:**
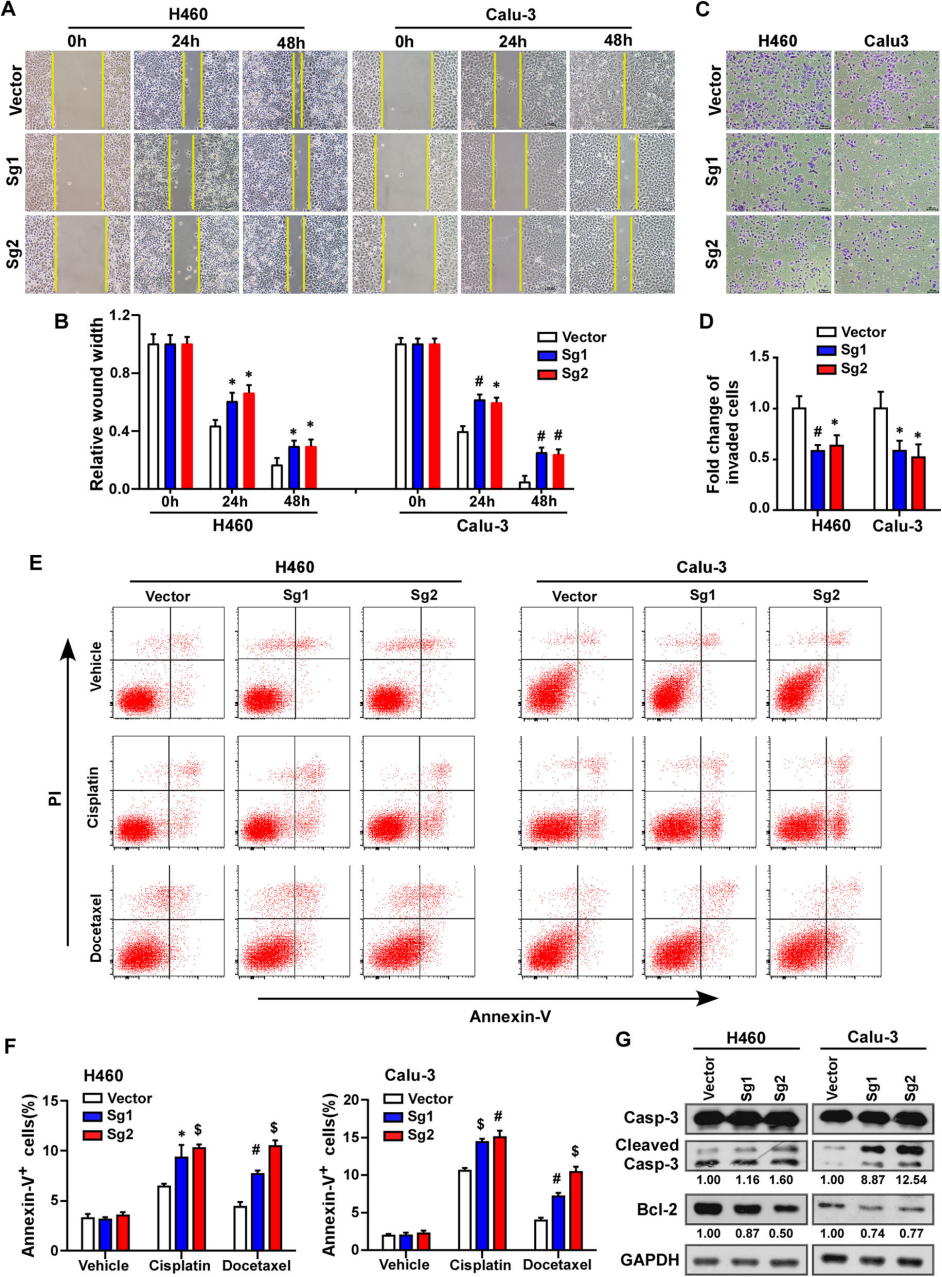
Depleting PTOV1 inhibits migration and invasion and promotes cell apoptosis. **a** and **b** Representative images (**a**) and quantification (**b**) of cell migration the indicated cells analyzed by wound healing assay. **c** and **d** Representative images (**c**) and quantification (**d**) of invade cells analyzed by chamber invasion assay. **e** and **f** Representative images (**e**) and quantification (**f**) of cell apoptosis of the indicated cells treated with or without drugs analyzed by Annexin V/PI staining. **g** Western blotting analysis of Caspase-3 (Casp-3), Cleaved Casp-3 and Bcl-2. GAPDH served as a loading control. Error bars represent mean ± SD from least 3 independent experiments. *, *P* < 0.05; #, *P* < 0.01; $, *P* < 0.001, two-tailed, unpaired t-test

Next, the effect of PTOV1 on tumor cell migration and invasion, which had been reported associating with chemo-resistance, were investigated. Depletion of PTOV1 slowed down the closure of a “wound” scratched into a confluent epithelial monolayer (Fig. 4a and b). Trans-well assay showed that both the migratory and invasive abilities of H460 and Calu3 cells with silenced PTOV1 expression was significantly reduced, as there were less cells migrated (Additional file 3: Figure S2A and B) or invaded (Fig. 4c and d) into the lower-surface of the membrane in the chamber. EGFP tagged cells analyzed under a fluorescent microscope further confirmed the results (Additional file 3: Figure S2C-F). Then, the effect of PTOV1 on cell apoptosis was analyzed. Flow cytometry assay revealed that there were much more Annexin V positive cells, indicating apoptotic cells, in PTOV1 knockout cells after drug treatment (Fig. 4e). Western blotting showed that the pro-apoptotic protein caspase-3 was activated, while the anti-apoptotic protein Bcl-2 was decreased, in PTOV1 depleted cells (Fig. 4f). Flow cytometry analysis also showed increased cleaved caspase-3 expression while depleting PTOV1 (Additional file 4: Figure S3). These findings proved that depletion of PTOV1 promoted chemotherapy-induced cell apoptosis.

Additionally, overexpressing PTOV1 in lung bronchus epithelial cell line BEAS-2B decreased the percentage of apoptotic cells induced by cisplatin and docetaxel analyzed by Annexin V/PI staining, which indicted decreased chemosensitivity of BEAS-2B/PTOV1 cells (Additional file 5: Figure S4A-C). Malignant transformation is an important aspect in cancer initiation. We then performed anchorage-independent cell growth of cells on soft agar to determine whether PTOV1 transforming BEAS-2B cells. However, neither BEAS-2B vector cells nor PTOV1 overexpressed cells formed any colonies larger than 50 μm in diameter (Additional file 5: Figure S4D, cells indicated by red arrows). Hence, we draw a conclusion that PTOV1 is crucial in drug resistance but might not in tumor initiation in NSCLC.

### Depleting PTOV1 attenuated stem cell-like properties

**Fig. 5:**
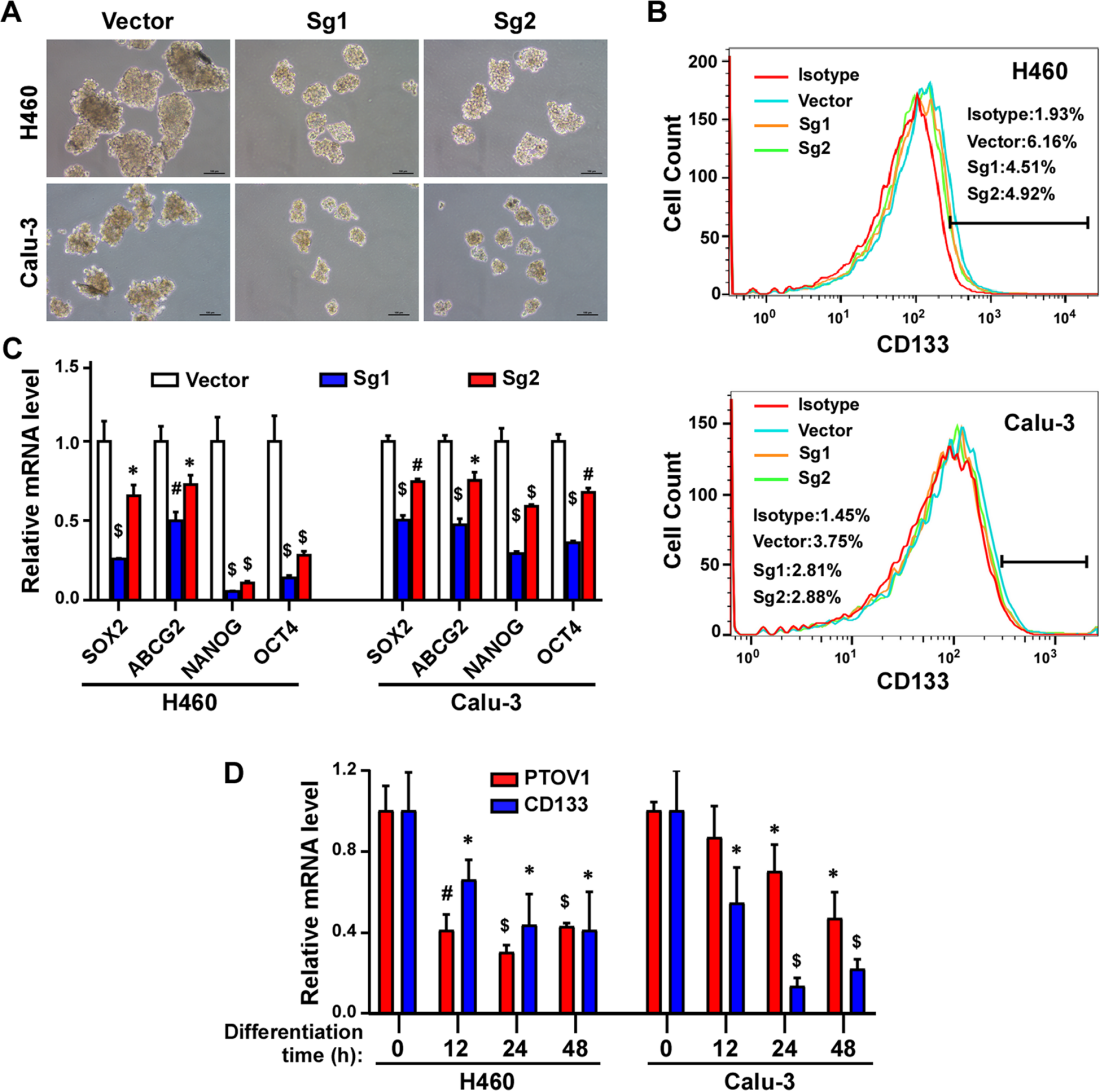
Depleting PTOV1 impaired stem cell-like properties in NSCLC cells. **a** Representative images of tumor sphere formation of the indicated cells. **b** FACS analysis of the portion of CD133^+^ cells. **c** Real-time quantitative PCR detecting the expression of the pluripotency factors in the indicated cells. **d** Real-time quantitative PCR detecting the expression of PTOV1 and CD133 in undifferentiated and serum-induced differentiated cells. Error bars represent the mean ± SD from 3 independent experiments. *, *P* < 0.05; #, *P* < 0.01; $, *P* < 0.001, two-tailed, unpaired t-test

Cancer stem cells (CSCs) or stem cell-like properties of cancer cells were reported contributing to cancer treatment failure/resistance and recurrence. Here we found that high PTOV1 level correlated with poor survival of NSCLC patients who received chemotherapy (Fig. 2c-e), indicating that PTOV1 might lead to chemoresistance. And LUAD patients with high PTOV1 got a short FPS time (Additional file 2: Figure S1C). These findings suggested that PTOV1 might associate with stemness in NSCLC. Indeed, in other models, PTOV1 was reported regulating stemness of tumor cells [21]. Thus, we explored whether PTOV1 also regulates the stemness of NSCLC cells. Tumor sphere formation assays showed that depleting PTOV1 significantly reduced the size and number of spheres than that formed by the vector cells (Fig. 5a and Additional file 6: Figure S5A). Flow cytometry analysis of the CSCs marker CD133 showed that silencing PTOV1 decreased CD133^+^ cell populations comparing to the vector control (Fig. 5b and Additional file 6: Figure S5B). Moreover, the mRNA expression levels of pluripotency factors, including SOX2, ABCG2, NANOG and OCT4, were also dramatically decreased in PTOV1 knockout cells (Fig. 5c). In further experiment, the expression of PTOV1 was analyzed during the differentiation process induced by serum exposure of tumor spheroids. As expected, PTOV1 mRNA decreased after differentiation induction, the same as the CSCs marker CD133 (Fig. 5d). Consistently, in the published microarray data, the expression of PTOV1 was higher in undifferentiated spheroids than that in differentiated cells (Additional file 6: Figure S5C and D). These results proved that PTOV1 plays important roles in stemness maintenance of NSCLC.

### Depleting PTOV1 attenuated stem cell-like properties via DKK1/β-catenin signaling

**Fig. 6:**
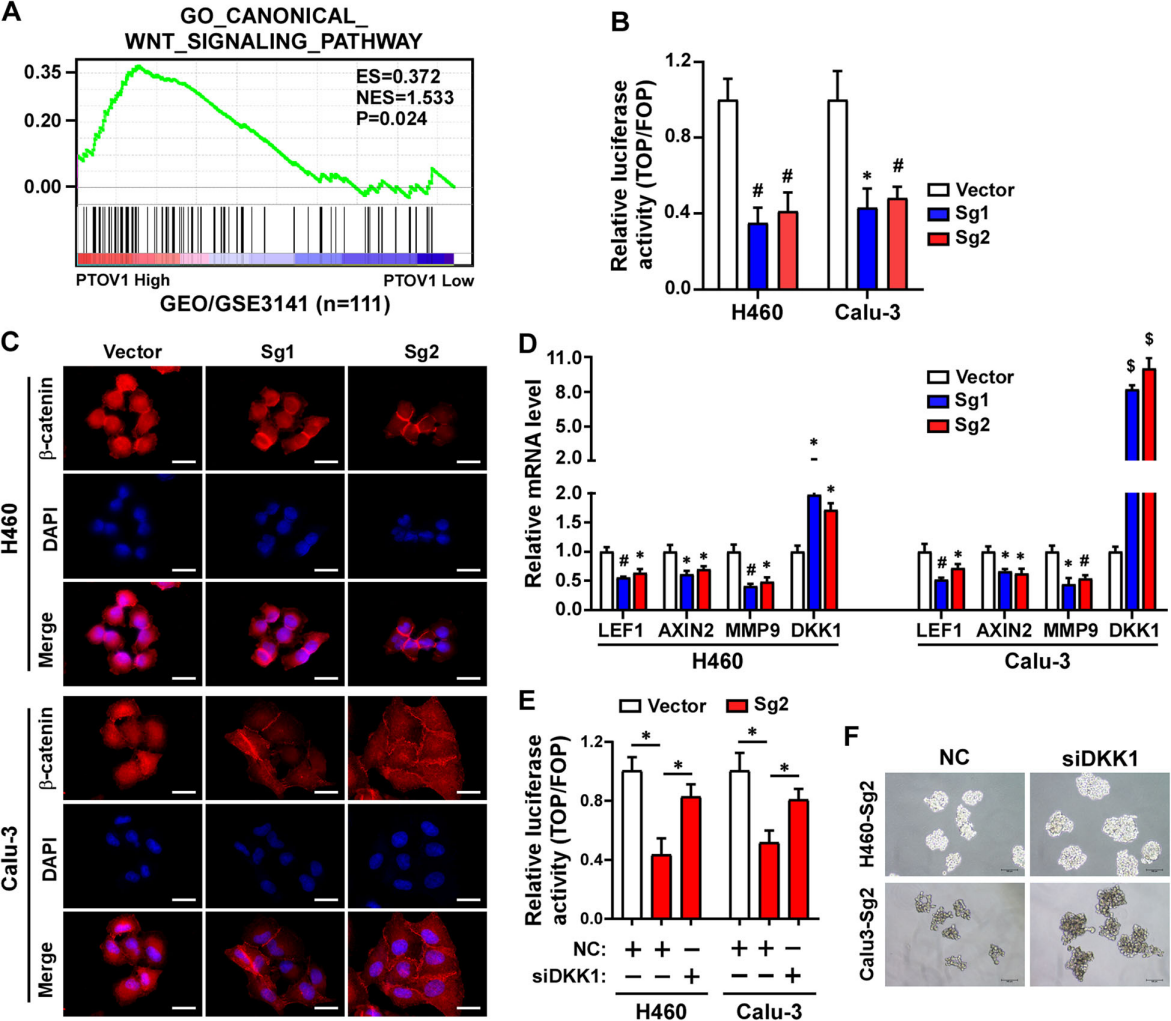
Depleting PTOV1 impaired stem cell-like properties via DKK1/β-catenin signaling. **a** GSEA analysis of the correlation between PTOV1 expression and the GO_ CANONICAL_WNT_SIGNALING_PATHWAY gene signature. **b** TOP/FOP Flash activity of the indicated cells. **c** Representative images of immunofluorescence staining of β-catenin in the indicated cells. **d** Real-time quantitative PCR detecting the expression of LEF1, AXIN2, MMP9and DKK1 in the indicated cells. **e** TOP/FOP Flash activity of the indicated cells transfected with NC or DKK siRNA. **f** Representative images of tumor sphere formation of the indicated cells transfected with NC or DKK siRNA. Error bars represent the mean ± SD from 3 independent experiments. *, *P* < 0.05; #, *P* < 0.01; $, *P* < 0.001, two-tailed, unpaired t-test

Next, we investigated which pathway involved in PTOV1 regulated CSCs. Gene enrichment analysis (GSEA) analyses revealed that PTOV1 expression positively correlated with the activation of the Wnt/β-catenin (Fig. 6a). TOP/FOP Flash activity was dramatically decreased in PTOV1 depleted cells (Fig. 6b). Immunofluorescence staining and nuclear protein extraction showed that depleting PTOV1 decreased nuclear localization of β-catenin protein (Fig. 6c and Additional file 7: Figure S6A). The expression of LEF1, AXIN2 and MMP9, the putative downstream genes of β-catenin, were also inhibited after silencing PTOV1 expression (Fig. 6d). These data proved that depleting PTOV1 impaired β-catenin.

It was previously reported that PTOV1 cloud impaired the expression of DKK1, a negative regulator of β-catenin, to activate β-catenin signaling in breast cancer [21]. Consistently, we found that DKK1 was upregulated in PTOV1 depleted cells (Fig. 6d and Additional file 7: Figure S6B). To determine whether upregulated DKK1 accounting for impaired β-catenin signaling activity in PTOV1 depleted cells, a siRNA specifically targeting DKK1 was used to inhibit DKK1 expression (Additional file 7: Figure S6C). Luciferase reporter assay showed that siDKK1 restored β-catenin transcriptional activity in PTOV1 depleted cells as indicated by TOP/FOP-Flash activity (Fig. 6e). What’s more, tumor sphere formation assay showed siDKK1 increased tumor sphere formation ability of PTOV1-Sg2 cells (Fig. 6f and Additional file 7: Figure S6D), which proved that inhibiting DKK1 restored stemness of PTOV1-depleted cells. Overall, we conclude that depleting PTOV1 upregulates DKK1, which further leads to impaired β-catenin signaling and stemness of NSCLC cells. These results suggested that depleting PTOV1 attenuated stem cell-like properties of NSCLC cells by inhibiting β-catenin signaling activation.

### Inhibition of PTOV1 chemosensitizes NSCLC cells in vivo

**Fig. 7:**
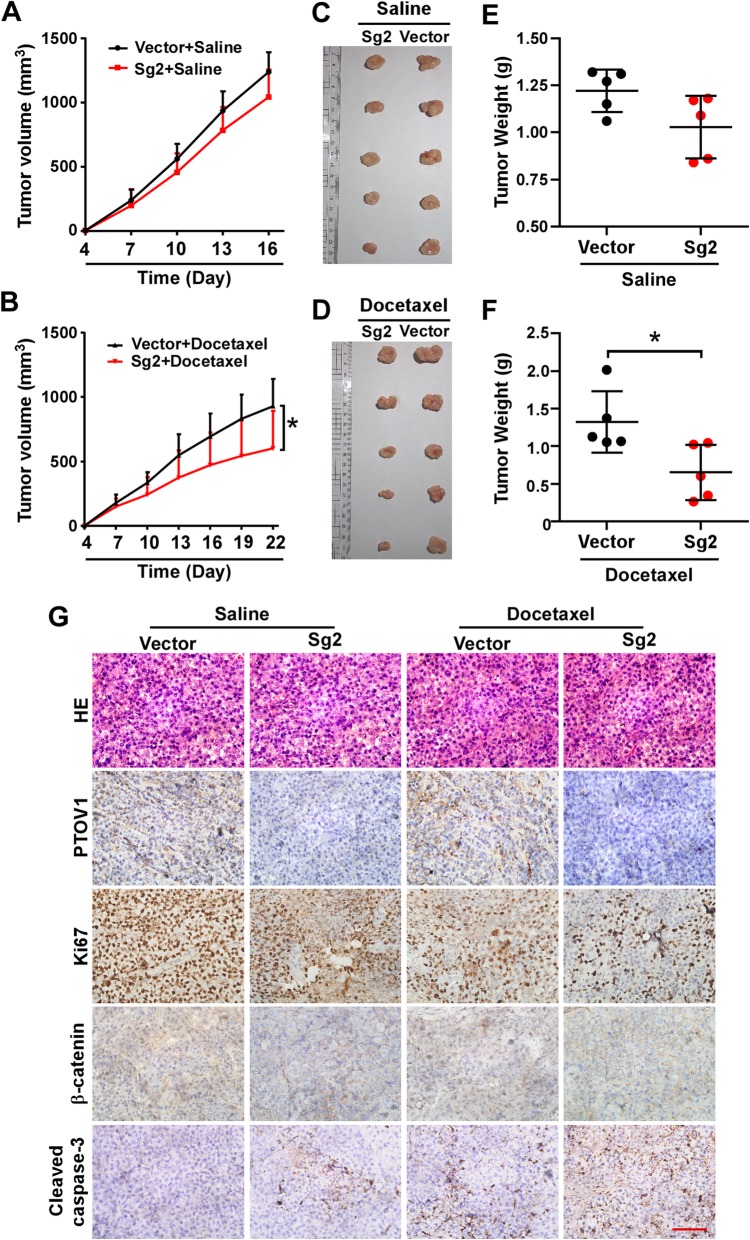
Depleting PTOV1 chemosensitizes NSCLC cells in vivo. **a** and **d** Growth curve of the tumor volumes measured on the indicated days. Error bars represent the mean ± SD. *, *P* < 0.05, one-way ANOVA test. **b** and **e** Representative pictures of tumor growth. **c** and **f** Quantification of tumor weights. Error bars represent the mean ± SD. *, *P* < 0.05, two-tailed, unpaired t-test. **g** Representative pictures of H&E staining and IHC staining of PTOV1, Ki-67, β-catenin and cleaved Caspase-3 in the indicated xenografted tumors. Scale bar, 50 μm

Lastly, we verified inhibiting PTOV1 sensitizing NSCLC to chemotherapy in vivo using H460 xenograft tumor model (Additional file 8: Figure S7). Depleting PTOV1 only slightly inhibited tumor growth as indicated by the tumor volume and weight when receiving no treatment (Fig. 7a, c, e). However, when treated with docetaxel, depleting PTOV1 significantly reduced tumor growth compared with the vector group (Fig. 7b, d, f). Furthermore, IHC staining showed that depleting PTOV1 significantly repressed expression of Ki-67, inhibited of β-catenin nuclear localization and increased expression of cleaved caspase-3 (Fig. 7g). Taken together, these results indicated that depleting PTOV1 inhibited cell proliferation, promoted cell apoptosis and sensitized NSCLC to chemotherapy in vivo.

## Discussion

PTOV1 was firstly identified and reported to be overexpressed in prostate cancer [14]. Later, it was found that high levels of PTOV1 correlated with poor prognosis of prostate cancer [35], breast cancer [20], urothelial carcinoma [36] and so on. Using tissue microarrays, Sara et al. unveiled high expression of PTOV1 in lung cancer [15]. However, the significance of PTOV1 expression in lung cancer has not been studied. Upon analyzing data of multiple microarrays from the NCBI/GEO and TCGA database, we found that PTOV1 was consistently upregulated in tumor tissues of NSCLC comparing to the normal control. Moreover, we performed IHC and statistical analyses in a cohort of NSCLC samples. Results showed that PTOV1 was also an independent and poor prognosis factor for NSCLC. Patients with high PTOV1 level got a shorter survival time than the ones with low PTOV1.

Recently, Verónica et al. reported that transduction of PTOV1 in Du145 and PC3 cells significantly increased cell survival after docetaxel exposure and induced docetaxel-resistance genes expression [37]. Previously, HyeSook Youn et al. revealed that PTOV1 cooperates with Zyxin for the negative regulation of RA signaling. RA-induced cancer cell cytotoxicity was significantly impaired by PTOV1 [33]. We therefore anticipated that PTOV1 may regulate chemotherapy sensitivity in NSCLC. By analyzing the subgroup NSCLC patients who received chemotherapy, we found that high PTOV1 also associated with short survival time. Surprisingly, the hazard ratio of high PTOV1 in NSCLC patients received chemotherapy is much higher than that in the pooled all patients, which indicated that PTOV1 could not only be a prognosis marker for NSCLC patients but also be better in predicting outcome to chemotherapy. Indeed, when depleting PTOV1 expression, we found that less cells survived after cisplatin and docetaxel exposure, and the IC50s of H460 and Calu-3 to these drugs were significantly decreased. Together, these findings suggest that PTOV1 regulates chemosensitivity in malignant tumors and depleting PTOV1 chemosensitizes NSCLC cells.

CSCs is a small subsets of cells capable of self-renewal and long-term repopulation. Evidences have proved that CSCs contribute to tumor recurrence, metastasis and treatment failure. In prostate cancer cells, transduction of PTOV1 induced prostatospheres formation and self-renewal genes expression [37]. Yanmei Cui et al. reported that, in breast cancer, PTOV1 enhanced CSCs population by recruiting HDAC1/2 to reduce DKK1 promoter histone acetylation and subsequently activating Wnt/β-catenin signaling [21]. Consistent with previous study, we found that PTOV1 also regulates CSCs or stem cell-like properties of NSCLC. Depleting PTOV1 impaired tumor sphere formation, reduced CD133^+^ cell population and decreased the expression of pluripotency factors of NSCLC cells. GSEA analysis showed that PTOV1 expression correlated with the activation of canonical Wnt signaling and Notch signaling (Fig. 5 and data not shown), which are all reported enhancing CSCs. However, it was previously reported that PTOV1 counteract the transcriptional activity of Notch [17], which is controversial to our results. Here we proved that PTOV1 regulates CSCs via DKK1/β-catenin signaling in NSCLC as reported [21]. Whether the Notch signaling contributing to the effects of PTOV1 on CSCs in NSCLC needs further investigation.

In conclusion, this study revealed PTOV1 as a poor prognosis factor for NSCLC patients, and targeting PTOV1 can be a strategy to increase chemosensitivity in NSCLC.

## Conclusion

In summary, we have demonstrated that PTOV1 is upregulated in NSCLC and its expression negatively correlates with prognosis of NSCLC patients. Our study provides compelling evidence that depleting PTOV1 can increase cell apoptosis, inhibit invasion, migration, stemness and tumorigenicity and chemosensitize NSCLC cells. These results suggest that PTOV1 is a potential prognostic and therapeutic biomarker for NSCLC.

## Additional files

Additional file 1: **Table S1.** Clinicopathological characteristics and the correlation between PTOV1 expression and the clinicopathological characteristics of NSCLC patients. **Table S2.** Sequences of real-time PCR primers. (DOCX 25 kb)

Additional file 2: **Figure S1.** PTOV1 associates with poor prognosis in NSCLC. (A and B) Kaplan–Meier analysis of overall survival. (C) Kaplan–Meier analysis of the first progression survival. (TIF 151 kb)

Additional file 3: **Figure S2.** Depleting PTOV1 impairs migration and invasion of NSCLC cells. (A and B) Representative images and quantification of migrated cells. (C and D) Representative images and quantification of wound healing assay using eGFP tagged cells. (E and F) Representative images and quantification of migrated and invaded cells using eGFP tagged cells. At least three independent experiments were performed. *, *P*<0.05; #, *P*<0.01; $, *P*<0.001. (TIF 2060 kb)

Additional file 4: **Figure S3.** Depleting PTOV1 activates caspase-3. Representative pictures (A) and quantification (B) of cleaved caspase-3. At least three independent experiments were performed. *, *P*<0.05; #, *P*<0.01; $, *P*<0.001. (CVX 347 kb)

Additional file 5: **Figure S4.** Overexpressing PTOV1 decreased chemosensitivity of BEAS-2B cells. (A and B) Immunoblotting and Q-PCR analyses of PTOV1. (C) Quantification of cell apoptosis. (D) Representative images of anchorage-independent cell growth. Red arrows indicate cells. At least three independent experiments were performed. *, *P*<0.05; $, *P*<0.001. (TIF 697 kb)

Additional file 6: **Figure S5.** PTOV1 level associates with CSCs properties. (A) Quantification of tumor spheres. (B) Quantification of CD133^+^ cells. (C) PTOV1 mRNA level in undifferentiated and serum-induced differentiation of H460 cell spheroids in the NCBI/GEO/GSE54712 dataset. (D) PTOV1 mRNA level detected. *P*<0.001. (TIF 181 kb)

Additional file 7: **Figure S6.** PTOV1 modulates DKK1/β-Catenin signaling. (A) Immunoblotting analysis of β-catenin. Lamin B1 and GAPDH are the nuclear and cytoplasmic marker respectively. (B) Immunoblotting analysis of DKK1. (C) Q-PCR and immunoblotting analyses of DKK1. GAPDH is loading control. (D) Quantification of tumor spheres. At least three independent experiments were performed. *, *P*<0.05; #, *P*<0.01. (TIF 221 kb)

Additional file 8: **Figure S7.** Schedule of xenograft tumor model. (TIF 31 kb)

## Data Availability

The datasets used and/or analyzed during the current study are available from the corresponding author on reasonable request.

## Abbreviations

CSCs: Cancer stem cells
GAPDH: Glyceraldehyde-3-phosphate dehydrogenase
GSEA: Gene set enrichment analysis
LUAD: Lung adenocarcinoma
LUSC: Lung squamous cell carcinoma
NSCLC: Non-small cell lung cancer
PTOV1: Prostate tumor over expressed gene 1
sgRNA: Small guide RNA

## References

[1] Torre LA, Bray F, Siegel RL, Ferlay J, Lortet-Tieulent J, Jemal A (2015). Global cancer statistics, 2012. CA Cancer J Clin.

[2] Siegel RL, Miller KD, Jemal A (2016). Cancer statistics, 2016. CA Cancer J Clin.

[3] Arriagada R, Bergman B, Dunant A, Le Chevalier T, Pignon JP, Vansteenkiste J (2004). Cisplatin-based adjuvant chemotherapy in patients with completely resected non-small-cell lung cancer. N Engl J Med.

[4] Winton T, Livingston R, Johnson D, Rigas J, Johnston M, Butts C (2005). Vinorelbine plus cisplatin vs. observation in resected non-small-cell lung cancer. N Engl J Med.

[5] Chang A (2011). Chemotherapy, chemoresistance and the changing treatment landscape for NSCLC. Lung Cancer.

[6] Clarke MF, Dick JE, Dirks PB, Eaves CJ, Jamieson CH, Jones DL (2006). Cancer stem cells--perspectives on current status and future directions: AACR workshop on cancer stem cells. Cancer Res.

[7] Plaks V, Kong N, Werb Z (2015). The cancer stem cell niche: how essential is the niche in regulating stemness of tumor cells?. Cell Stem Cell.

[8] Eramo A, Lotti F, Sette G, Pilozzi E, Biffoni M, Di Virgilio A (2008). Identification and expansion of the tumorigenic lung cancer stem cell population. Cell Death Differ.

[9] Chen YC, Hsu HS, Chen YW, Tsai TH, How CK, Wang CY (2008). Oct-4 expression maintained cancer stem-like properties in lung cancer-derived CD133-positive cells. PLoS One.

[10] Chiou SH, Wang ML, Chou YT, Chen CJ, Hong CF, Hsieh WJ (2010). Coexpression of Oct4 and Nanog enhances malignancy in lung adenocarcinoma by inducing cancer stem cell-like properties and epithelial-mesenchymal transdifferentiation. Cancer Res.

[11] Donnenberg VS, Donnenberg AD (2005). Multiple drug resistance in cancer revisited: the cancer stem cell hypothesis. J Clin Pharmacol.

[12] Gomez-Casal R, Bhattacharya C, Ganesh N, Bailey L, Basse P, Gibson M (2013). Non-small cell lung cancer cells survived ionizing radiation treatment display cancer stem cell and epithelial-mesenchymal transition phenotypes. Mol Cancer.

[13] Bertolini G, Roz L, Perego P, Tortoreto M, Fontanella E, Gatti L (2009). Highly tumorigenic lung cancer CD133+ cells display stem-like features and are spared by cisplatin treatment. Proc Natl Acad Sci U S A.

[14] Benedit P, Paciucci R, Thomson TM, Valeri M, Nadal M, Caceres C (2001). PTOV1, a novel protein overexpressed in prostate cancer containing a new class of protein homology blocks. Oncogene.

[15] Fernandez S, Mosquera JL, Alana L, Sanchez-Pla A, Morote J, Ramon YCS (2011). PTOV1 is overexpressed in human high-grade malignant tumors. Virchows Arch.

[16] Santamaria A, Fernandez PL, Farre X, Benedit P, Reventos J, Morote J (2003). PTOV-1, a novel protein overexpressed in prostate cancer, shuttles between the cytoplasm and the nucleus and promotes entry into the S phase of the cell division cycle. Am J Pathol.

[17] Alana L, Sese M, Canovas V, Punyal Y, Fernandez Y, Abasolo I (2014). Prostate tumor OVerexpressed-1 (PTOV1) down-regulates HES1 and HEY1 notch targets genes and promotes prostate cancer progression. Mol Cancer.

[18] Youn HS, Park UH, Kim EJ, Um SJ (2011). PTOV1 antagonizes MED25 in RAR transcriptional activation. Biochem Biophys Res Commun.

[19] Chen SP, Zhang LS, Fu BS, Zeng XC, Yi HM, Jiang N (2015). Prostate tumor overexpressed 1 is a novel prognostic marker for hepatocellular carcinoma progression and overall patient survival. Medicine (Baltimore).

[20] Lei F, Zhang L, Li X, Lin X, Wu S, Li F (2014). Overexpression of prostate tumor overexpressed 1 correlates with tumor progression and predicts poor prognosis in breast cancer. BMC Cancer.

[21] Cui Y, Ma W, Lei F, Li Q, Su Y, Lin X (2016). Prostate tumour overexpressed-1 promotes tumourigenicity in human breast cancer via activation of Wnt/beta-catenin signalling. J Pathol.

[22] Landi MT, Dracheva T, Rotunno M, Figueroa JD, Liu H, Dasgupta A (2008). Gene expression signature of cigarette smoking and its role in lung adenocarcinoma development and survival. PLoS One.

[23] Lu TP, Tsai MH, Lee JM, Hsu CP, Chen PC, Lin CW (2010). Identification of a novel biomarker, SEMA5A, for non-small cell lung carcinoma in nonsmoking women. Cancer Epidemiol Biomark Prev.

[24] Selamat SA, Chung BS, Girard L, Zhang W, Zhang Y, Campan M (2012). Genome-scale analysis of DNA methylation in lung adenocarcinoma and integration with mRNA expression. Genome Res.

[25] Hou J, Aerts J, den Hamer B, van Ijcken W, den Bakker M, Riegman P (2010). Gene expression-based classification of non-small cell lung carcinomas and survival prediction. PLoS One.

[26] Lopez-Ayllon BD, Moncho-Amor V, Abarrategi A, Ibanez de Caceres I, Castro-Carpeno J, Belda-Iniesta C (2014). Cancer stem cells and cisplatin-resistant cells isolated from non-small-lung cancer cell lines constitute related cell populations. Cancer Med.

[27] Ishiguro T, Sato A, Ohata H, Ikarashi Y, Takahashi RU, Ochiya T (2016). Establishment and characterization of an in vitro model of ovarian Cancer stem-like cells with an enhanced proliferative capacity. Cancer Res.

[28] Nagy A, Lanczky A, Menyhart O, Gyorffy B (2018). Validation of miRNA prognostic power in hepatocellular carcinoma using expression data of independent datasets. Sci Rep.

[29] Bild AH, Yao G, Chang JT, Wang Q, Potti A, Chasse D (2006). Oncogenic pathway signatures in human cancers as a guide to targeted therapies. Nature.

[30] Sanjana NE, Shalem O, Zhang F (2014). Improved vectors and genome-wide libraries for CRISPR screening. Nat Methods.

[31] Wu Z, Zhao J, Qiu M, Mi Z, Meng M, Guo Y (2018). CRISPR/Cas9 mediated GFP Knock-in at the MAP 1LC3B locus in 293FT cells is better for Bona fide monitoring cellular autophagy. Biotechnol J.

[32] Xia J, Wu Z, Yu C, He W, Zheng H, He Y (2012). miR-124 inhibits cell proliferation in gastric cancer through down-regulation of SPHK1. J Pathol.

[33] Youn H, Kim EJ, Um SJ (2013). Zyxin cooperates with PTOV1 to confer retinoic acid resistance by repressing RAR activity. Cancer Lett.

[34] Rinaldi M, Cauchi C, Gridelli C (2006). First line chemotherapy in advanced or metastatic NSCLC. Ann Oncol.

[35] Morote J, Fernandez S, Alana L, Iglesias C, Planas J, Reventos J (2008). PTOV1 expression predicts prostate cancer in men with isolated high-grade prostatic intraepithelial neoplasia in needle biopsy. Clin Cancer Res.

[36] Rausch S, Hennenlotter J, Scharpf M, Teepe K, Kuhs U, Aufderklamm S (2016). Prostate tumor overexpressed 1 expression in invasive urothelial carcinoma. J Cancer Res Clin Oncol.

[37] Canovas V, Punal Y, Maggio V, Redondo E, Marin M, Mellado B (2017). Prostate tumor Overexpressed-1 (PTOV1) promotes docetaxel-resistance and survival of castration resistant prostate cancer cells. Oncotarget.

