# Supplementary material for: Depleting PTOV1 sensitizes non-small cell lung cancer cells to chemotherapy through attenuating cancer stem cell traits
Source: J Exp Clin Cancer Res. 2019 Aug 6;38:341. doi: 10.1186/s13046-019-1349-y (PMC6685258; doi:10.1186/s13046-019-1349-y)
Supplement: Supplementary file 1 — Table S1. Clinicopathological characteristics and the correlation between PTOV1 expression and the clinicopathological characteristics of NSCLC patients. Table S2. Sequences of real-time PCR primers. (DOCX 25 kb) [file 13046_2019_1349_MOESM1_ESM.docx]

| **Characteristics** | | **PTOV1 expression** | | **χ^2^ Test P-Value** | **Fisher Exact**  **Test P-Value** |
| --- | --- | --- | --- | --- | --- |
|  |  | **Low** | **High** |  |  |
| **Sex** | Male | 37 | 27 | 0.226 | 0.236 |
|  | Female | 58 | 28 |  |  |
| **Age(years)** | ≥60 | 48 | 28 | 0.964 | 1.000 |
|  | ＜60 | 47 | 27 |  |  |
| **Smoke** | Yes | 40 | 24 | 0.855 | 0.866 |
|  | No | 55 | 31 |  |  |
| **T classification** | T1 | 59 | 31 | 0.668 | 0.644 |
|  | T2 | 34 | 21 |  |  |
|  | T3 | 1 | 2 |  |  |
|  | T4 | 1 | 1 |  |  |
| **N classification** | N0 | 58 | 30 | 0.580 | 0.589 |
|  | N1 | 5 | 5 |  |  |
|  | N2 | 32 | 20 |  |  |
| **M classification** | M0 | 92 | 55 | 0.183 | 0.299 |
|  | M1 | 3 | 0 |  |  |
| **Clinical stage** | Ⅰ | 55 | 29 | 0.449 | 0.499 |
|  | Ⅱ | 7 | 6 |  |  |
|  | Ⅲ | 30 | 20 |  |  |
|  | Ⅳ | 3 | 0 |  |  |
| **Chemotherapy** | Yes | 55 | 31 | 0.855 | 0.866 |
|  | No | 40 | 24 |  |  |
| **EGFR status** | WT | 46 | 33 | 0.171 | 0.180 |
|  | Mut | 49 | 22 |  |  |

**Additional file 1**

**Table S1**  Clinicopathological characteristics and the correlation between PTOV1 expression and the clinicopathological characteristics of NSCLC patients

**Table S2** Sequences of real-time PCR primers.

| **Primer name** | **Sequence** |
| --- | --- |
| PTOV1-F | CCCAGTCCAGATCGT CAACA |
| PTOV1-R | GCCACCTCTTGGACCGACT |
| ABCG2-F | CTGAGATCCTGAGCCTTTGG |
| ABCG2-R | AAGCCA TTGGTGTTTCCTTG |
| NANOG-F | GATTTGTGGGCCTGAAGAAA |
| NANOG-R | CAGGGCTGTCCTGAATAAGC |
| SOX2-F | AGAAAAAC GAGGGAAATGGG |
| SOX2-R | GTCATTTGCTGTGGGTGATG |
| OCT4-F | GAAGGAGAAGCTGGAGCAAA |
| OCT4-R | CATCGGCCTGTG TATATCCC |
| GAPDH-F | GAAGGTGAAGGTCGGAGTCA |
| GAPDH-R | TTGAGGTCAATGAAGGGGTC |
| LEF1-F | TGGATCTCTTTCTCCACCCA |
| LEF1-R | TCCTGGAGAAAAGTGCTCGT |
| AXIN2-F | AAGTGCAAACTTTCGCCAAC |
| AXIN2-R | ACAGGATCGCTCCTCTTGAA |
| MMP9-F | GGGACGCAGACATCGTCATC |
| MMP9-R | TCGTCATCGTCGAAATGGGC |
| DKK1-F | TTTCCTCAATTTCTCCTCGG |
| DKK1-R | ATGCGTCACGCTATGTGCT |
| CD133-F | GGCCCAGTACAACACTACCAA |
| CD133-R | ATTCCGCCTCCTAGCACTGAA |
