# Supplementary figures and images for: Depleting PTOV1 sensitizes non-small cell lung cancer cells to chemotherapy through attenuating cancer stem cell traits
Source: J Exp Clin Cancer Res. 2019 Aug 6;38:341. doi: 10.1186/s13046-019-1349-y (PMC6685258; doi:10.1186/s13046-019-1349-y)

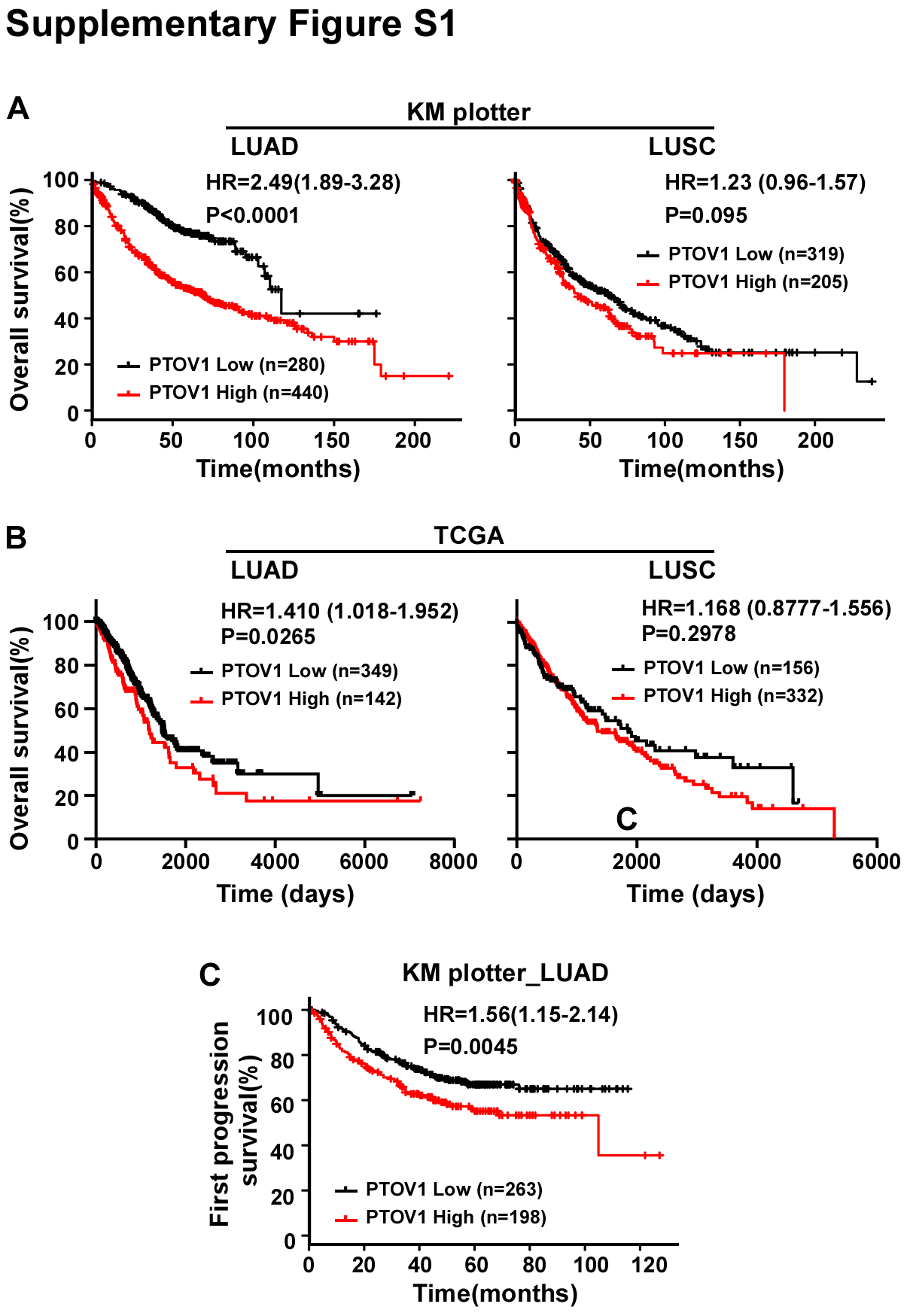

Supplement: Supplementary file 2 — Figure S1. PTOV1 associates with poor prognosis in NSCLC. (A and B) Kaplan–Meier analysis of overall survival. (C) Kaplan–Meier analysis of the first progression survival. (TIF 151 kb) [file 13046_2019_1349_MOESM2_ESM.tif]

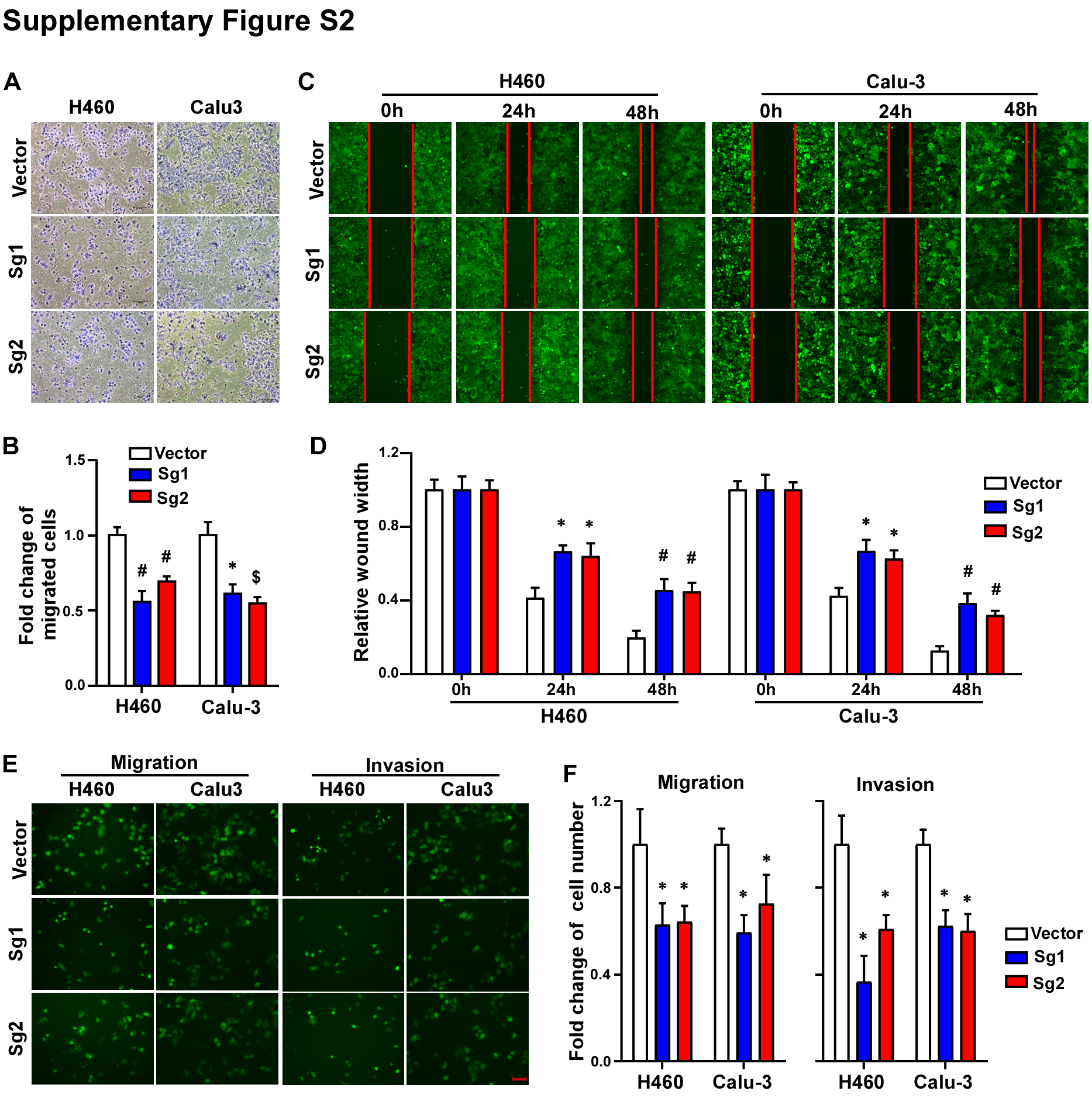

Supplement: Supplementary file 3 — Figure S2. Depleting PTOV1 impairs migration and invasion of NSCLC cells. (A and B) Representative images and quantification of migrated cells. (C and D) Representative images and quantification of wound healing assay using eGFP tagged cells. (E and F) Representative images and quantification of migrated and invaded cells using eGFP tagged cells. At least three independent experiments were performed. *, P<0.05; #, P<0.01; $, P<0.001. (TIF 2060 kb) [file 13046_2019_1349_MOESM3_ESM.tif]

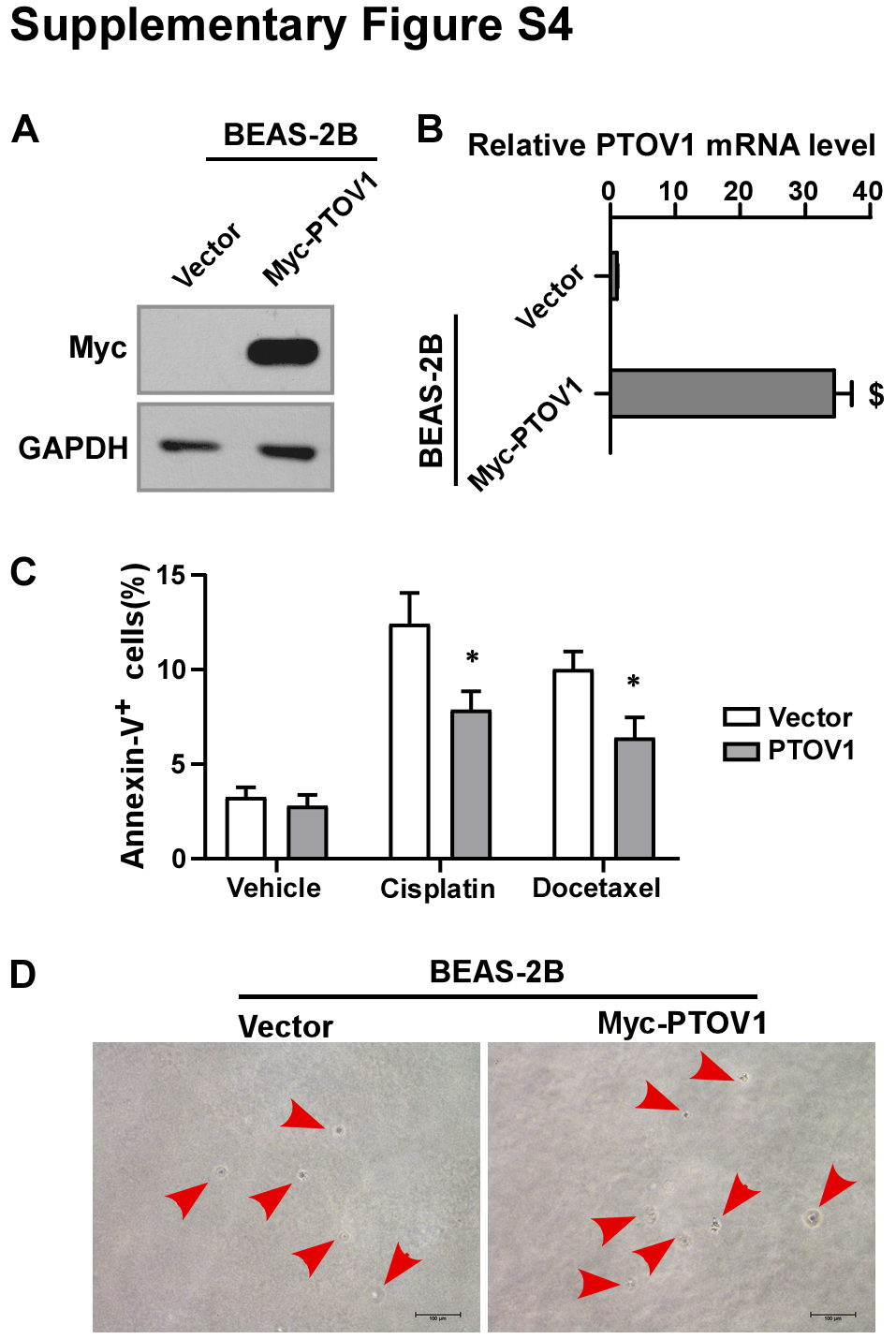

Supplement: Supplementary file 5 — Figure S4. Overexpressing PTOV1 decreased chemosensitivity of BEAS-2B cells. (A and B) Immunoblotting and Q-PCR analyses of PTOV1. (C) Quantification of cell apoptosis. (D) Representative images of anchorage-independent cell growth. Red arrows indicate cells. At least three independent experiments were performed. *, P<0.05; $, P<0.001. (TIF 697 kb) [file 13046_2019_1349_MOESM5_ESM.tif]

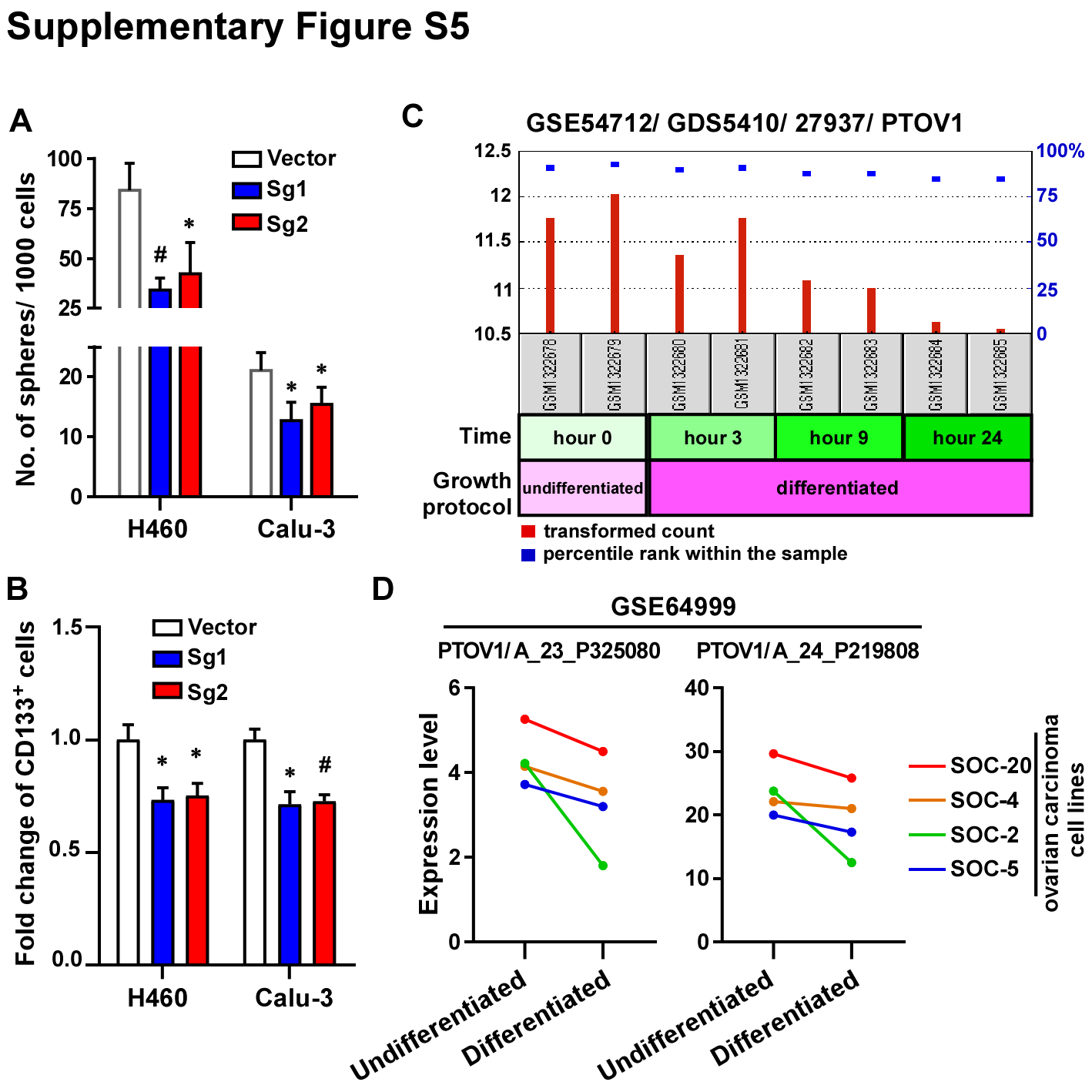

Supplement: Supplementary file 6 — Figure S5. PTOV1 level associates with CSCs properties. (A) Quantification of tumor spheres. (B) Quantification of CD133+ cells. (C) PTOV1 mRNA level in undifferentiated and serum-induced differentiation of H460 cell spheroids in the NCBI/GEO/GSE54712 dataset. (D) PTOV1 mRNA level detected. P<0.001. (TIF 181 kb) [file 13046_2019_1349_MOESM6_ESM.tif]

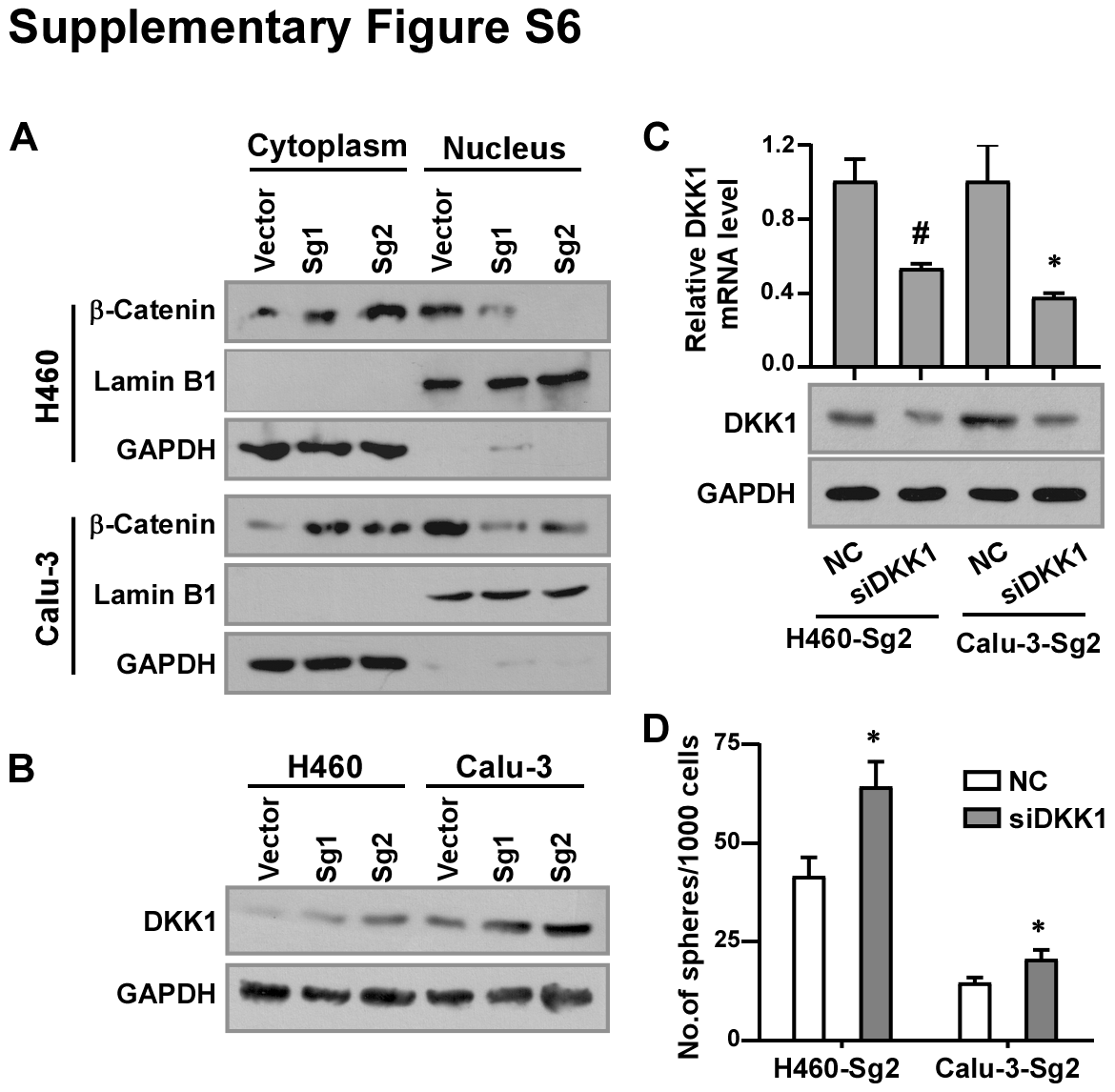

Supplement: Supplementary file 7 — Figure S6. PTOV1 modulates DKK1/β-Catenin signaling. (A) Immunoblotting analysis of β-catenin. Lamin B1 and GAPDH are the nuclear and cytoplasmic marker respectively. (B) Immunoblotting analysis of DKK1. (C) Q-PCR and immunoblotting analyses of DKK1. GAPDH is loading control. (D) Quantification of tumor spheres. At least three independent experiments were performed. *, P<0.05; #, P<0.01. (TIF 221 kb) [file 13046_2019_1349_MOESM7_ESM.tif]

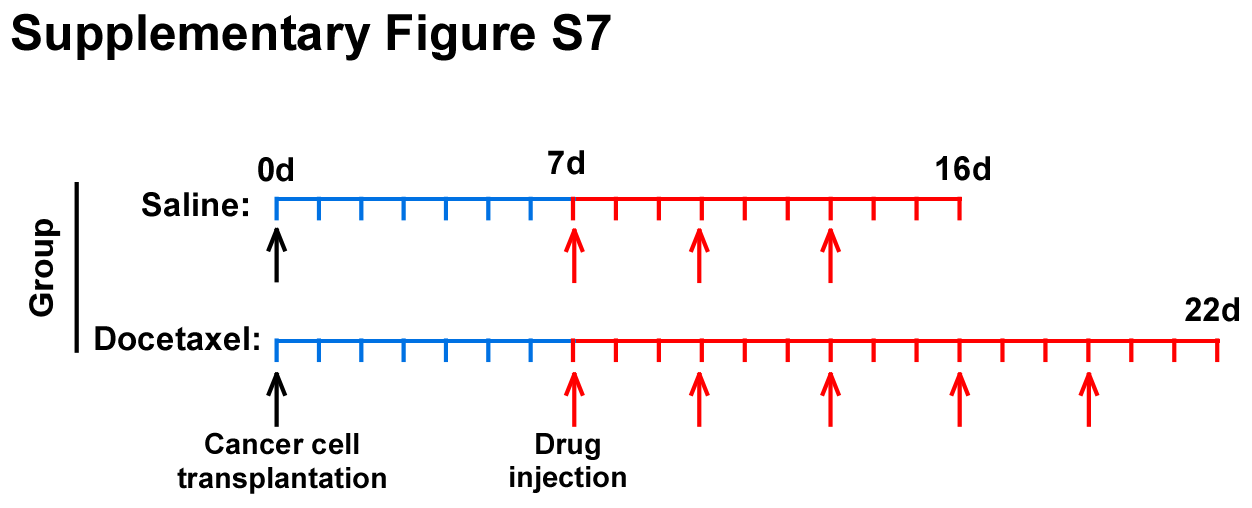

Supplement: Supplementary file 8 — Figure S7. Schedule of xenograft tumor model. (TIF 31 kb) [file 13046_2019_1349_MOESM8_ESM.tif]
